# Effects of dietary *Inonotus obliquus* fermentation product supplementation on growth performance, immune function, blood glucose level, and gut microbiota in cats

**DOI:** 10.3389/fvets.2026.1815007

**Published:** 2026-07-01

**Authors:** Lin Zhang, Xiaojie Gao, Junkun Wang, Wangwang Sun, Wenyu Cheng, Lifen Lian, Yubao Li

**Affiliations:** 1 Shandong Academy of Agricultural Sciences, Jinan, China; 2 Institute of Animal Science and Veterinary Medicine, Shandong Academy of Agricultural Sciences, Jinan, China; 3 Key Laboratory of Livestock and Poultry Multi-omics of MARA, Jinan, China; 4 College of Agriculture and Biology, Liaocheng University, Liaocheng, China; 5 Phage Research Center of Liaocheng University, Liaocheng, China

**Keywords:** blood glucose, cat, gut microbiome, *Inonotus obliquus* fermentation product, metabolome

## Abstract

*Inonotus obliquus* is a medicinal fungus rich in bioactive compounds that has demonstrated significant efficacy in animals when supplemented in the diet. However, its effects on key health parameters in cats remain unclear. This study evaluated the effects of *Inonotus obliquus* fermentation product (IOFP) as a functional preparation for cats. A total of 20 weaned kittens were divided into two groups, and each group was fed a basal diet or a diet supplemented with 0.8% IOFP (w/w) for 45 days. Growth performance, fasting blood glucose levels, and relative immune indicators were determined every 15 days. Post-trial intestinal samples were subjected to gut morphology, microbiota, and metabolomic analysis. Results indicated that IOFP supplementation significantly enhanced average daily gain and feed conversion efficiency, moderately lowered blood glucose levels, improved immune indicators, and reduced inflammatory cytokines. In addition, IOFP significantly increased gut microbial diversity and altered its composition. Metabolomic changes were consistent with anti-inflammatory and antitumor effects, with increased production of short-chain fatty acids, whereas metabolites linked to toxicity and metabolic disruption decreased. Pearson correlation analysis indicated that most metabolites were regulated by various bacteria. In conclusion, dietary IOFP supplementation improved growth performance and immunity, reduced blood glucose levels, and likely alleviated inflammation, probably by modulating the gut microbiota and its metabolites in cats, supporting its potential as a novel functional preparation for cats.

## 1 Introduction

As living standards continue to improve, people’s spiritual and cultural needs are growing increasingly. Pets, as animals close relationship with human life, have gradually shifted from their traditional roles in production and daily life to members of the family in modern society, primarily because of their emotional value (1). In recent years, the number of pets has been rising steadily. The domestic cat population in urban of China is projected to reach 72.89 million by 2025, and the number is expected to continue growing over a reasonable period of time (2). With the increasing public awareness of pet care, healthy and refined pet ownership became an inevitable trend, directly driving the industry’s transition from rapid expansion to high-quality development (3). Therefore, achieving both quantity and quality improvements in cats is essential for the healthy development of the industry.

The long-term use of antibiotics in animal husbandry had improved animal health and offered economic benefits for the industry. However, current emergencies, including environmental pollution, bacterial resistance, and drug residues, have raised public concern and prompted the search for viable alternatives (4–6). Natural substances and their biological agents, with green, safe, and effective characteristics, are extensively utilized in animal production. They play significant roles in production enhancement, disease reduction, and reduced use of antibiotics (7).

*Inonotus obliquus* (*I. obliquus*) is a perennial fungus that grows in cold regions and has long been used to treat various malignant and chronic diseases (8, 9). Modern medical research indicates that *I. obliquus* exhibits multiple biological activities, including antitumor, antioxidant, hypoglycemic, immunomodulatory, antibacterial, and antiviral effects (10, 11), asserting its significant therapeutic potential. However, its application in animals is starting. Our previous studies demonstrated that *I. obliquus* fermentation product (IOFP) supplemented in poultry diets significantly improved growth performance, immune function, and antioxidant capacity (12); when supplemented to mammals, IOFP could enhance the integrity and function of intestinal barrier and antioxidant capacity by modulating the gut microbiota and metabolites, which ultimately improved the growth performance, immune status, and meat quality (13, 14). These findings confirmed that IOFP was an effective feed additive and immunomodulator for poultry and mammals. Nevertheless, whether similar effects can be achieved in pet cats remains to be examined. In addition, cat ownership has surged since 2018, and there is a substantial population of older cats until now, in which metabolic disorders such as abnormal blood glucose levels and obesity are highly prevalent (15, 16). Whether *I. obliquus,* known for its blood glucose–regulation properties, could modulate glycemic levels is another noteworthy subject. Thus, in this study, IOFP was supplemented in the cat diet, and its effects on production performance, immune function, blood glucose levels, and the gut microbiota were comprehensively assessed. These findings may aid in the development of novel, green biological agents suitable for pets.

## 2 Materials and methods

### 2.1 Preparation of IOFP

IOFP was prepared by Qinhuangdao Gaotong Biotechnology Co., Ltd., in accordance with the method described in our previous study (14).

### 2.2 Animals and experimental design

A total of 20 healthy weaned Linqing Lion cats (age: 45–50 days) with similar body weights were purchased from the Linqing Lion Cat Conservation and Breeding Base. The cats were randomly assigned to two groups with 5 replicates in each group (two cats per replicate), ensuring equal distribution of males and females. The control group was fed a basal diet (formulated by Shandong Kuanfu Pet Food Co., Ltd. with reference to GB/T 31217–2014; the specific composition and nutritional levels are depicted in Table 1), whereas the IOFP group received the same commercial diet supplemented with IOFP (0.8% w/w). Each cat was housed individually with free access to food and water throughout the 45-day experimental period. The feed intake, mental state, and clinical symptoms were monitored daily.

**Table 1:** Ingredients and nutrient composition of the basal diet fed to the experimental cats.

Raw material components	Content	Nutrient level	Content
Chicken meal/%	38	Crude Protein/%	31.84
Duck meal/%	10	Crude Ash/%	8.1
Beef heart meal/%	8	Crude Fiber/%	4.2
Corn meal/%	10	Crude Fat/%	17.8
Chicken oil/%	15	Ca/%	1.39
Fish oil/%	8	Moisture/%	4.2
Sugar beet pulp/%	2	Water-soluble Chloride/%	0.6
Broccoli meal/%	1.75	TP/%	1.19
Marigold meal/%	1.5	Taurine/%	0.44
Blueberry meal/%	0.75		
Cranberry meal/%	0.5		
Calcium bicarbonate/%	2		
Sodium chloride/%	0.5		
Premix/%	2		
Total/%	100		

Ca, calcium; TP, total phosphorus.

### 2.3 Data record and sample collection

The body weight and feed consumption of the cats in both groups were weighed at day 0 and every 15-day intervals, which determined the average daily gain (ADG), average daily feed intake (ADFI), and feed-to-gain ratio (F/G). At the same timepoints, peripheral blood samples, used to determine fasting blood glucose levels and relevant immune parameters were collected. At the end of the 45-day trial, the cats were euthanized in accordance with the previously established guidelines (17). Approximately 2-cm segments from different regions of the small intestine (duodenum, jejunum, and ileum) were collected and fixed in 4% tissue fixative for histological sectioning. The cecal contents were collected for the analysis of gut microbiota, metabolites, and short-chain fatty acids (SCFAs), which conducted by Novogene Co., Ltd., based on the Illumina PE250, Thermo Q Exactive™ HF-X, and Thermo Scientific™ TSQ Altis™ platforms, respectively.

### 2.4 Determination of blood glucose levels in cats

The blood glucose levels were determined as the method described previously (18). All measurements were expressed in millimoles per liter (mmol/L) for subsequent statistical analyses.

### 2.5 Determination of serum immunoglobulin and cytokine

The concentrations of immunoglobulin A (IgA), IgG, IgM, interleukin-2 (IL-2), IL-4, IL-6, IL-10, interferon-*γ* (IFN-γ), tumor necrosis factor-*α* (TNF-α), and T-cell subsets (CD4^+^ and CD8^+^) were determined using enzyme-linked immunosorbent assay (ELISA) kits (Enzyme-linked Biotechnology Co., Ltd., Shanghai, China).

### 2.6 Preparation of tissue sections

The tissue sections were prepared as the description in a previous study (19). Briefly, three intact intestinal villi and corresponding crypts were selected and determined from each tissue section. The ratio of the villus height to crypt depth (V/C) was calculated, and the results were expressed as the mean ± standard error of the mean (SEM).

### 2.7 Analysis of gut microbiota via 16S rRNA sequencing

The cecal contents were harvested from 10 animals of each group for microbiome sequencing. Total genomic DNA was isolated from each specimen by using the QIAamp DNA Stool Mini Kit (Qiagen, Valencia, CA, United States), with strict adherence to the recommended protocol. Following quantification of the DNA yield, the samples were normalized to a final concentration of 1 ng/μL using nuclease-free distilled water. For library preparation, the hypervariable V3–V4 domains of the bacterial 16S ribosomal RNA gene were targeted and amplified with locus-specific oligonucleotide primers. The amplicon libraries were subjected to paired-end sequencing on an Illumina platform by Novo Gene Co., Ltd. (Beijing, China).

Bioinformatics processing of the raw sequencing data was conducted as follows: Overlapping paired-end reads were initially assembled into contiguous sequences using FLASH (v1.2.7). Quality-based trimming and length filtering were subsequently applied, followed by the elimination of PCR artifacts and chimeric amplicons using the QIIME pipeline (v1.9.1). High-fidelity sequences were subjected to operational taxonomic unit (OTU) delineation at a 97% pairwise identity cutoff via the Uparse algorithm (v7.0.1001). Taxonomic classification of the representative OTU sequences was performed by alignment against the SILVA release 138 SSU rRNA reference database using the Mothur software suite. Microbial diversity metrics were computed from the OTU abundance matrix to assess the within-group microbial richness and evenness.

### 2.8 Determination of cecal content metabolome

Cecal digesta specimens were subjected to comprehensive metabolic profiling using liquid chromatography coupled with tandem mass spectrometry (LC–MS/MS). The analytical workflow was sequentially executed, beginning with exhaustive metabolite recovery through solvent-based extraction, followed by chromatographic separation and mass spectrometric detection utilizing ultra-high performance liquid chromatography–tandem mass spectrometry (UHPLC–MS/MS) for quantitative metabolite assessment in the cecal matrix, culminating in computational data processing coupled with structural elucidation of the detected compounds. Metabolic pathway mapping and functional categorization of the identified molecules were executed through the interrogation of the Kyoto Encyclopedia of Genes and Genomes (KEGG) repository. Statistically significant differential metabolites were determined by applying stringent selection thresholds of absolute log2-fold change exceeding 1.0, and statistical significance was set at *p* < 0.05.

### 2.9 Determination of SCFAs in cecal contents of cats

Approximately 50 mg of the cecal content sample was weighed, and 500 μL of 80% methanol was added to precipitate the proteins therein. Two steel beads were added, and the mixture was homogenized by bead beating for 20 min, followed by centrifugation for 15 min (4 °C, 12,000 rpm). A 20 μL aliquot of the supernatant was transferred into a new 1.5-mL EP tube. Subsequently, the EDC solution and 3-NPH were added for derivatization, followed by the addition of the initial mobile phase solution upto a final volume of 500 μL. The mixture was then vortexed thoroughly, and the supernatant was collected for LC–MS/MS analysis.

### 2.10 Statistical analysis

Statistical analysis was performed using GraphPad Prism software (version 8.3), and all experimental data are presented as SEM. Differences between the groups were assessed using the independent samples t-test and one-way analysis of variance (ANOVA), followed by Duncan’s multiple comparison test. *p* < 0.05 was considered to indicate statistical significance.

## 3 Results

### 3.1 Effects of IOFP on the growth performance of cats

The growth performance of cats in the two groups was initially evaluated. As listed in Table 2, although the initial body weights of cats in the two groups were similar, the final body weight of the IOFP group was significantly higher than that of the control group (*p* < 0.05). Specifically, compared with the control group, increased ADG, and decreased ADFI (except during the 16–30-day period) in the IOFP group during the experimental period were observed. Consequently, F/G was significantly reduced in the IOFP group. In summary, dietary supplementation with IOFP significantly enhanced the growth performance of cats.

**Table 2:** Effect of *Inonotus obliquus* fermentation product (IOFP) on cat productivity.

Item	IOFP^1^	Control^2^	SEM	*P-*value
Initial BW, g	938.67	943.21	31.58	0.893
Final BW, g	1784.57	1587.11	25.97	0.02
d 1–15				
ADFI (g/d)	60.81^a^	66.26^b^	0.79	0.02
ADG (g/d)	16.89^a^	12.15^b^	0.58	0.01
F/G	3.60^a^	5.47^b^	0.19	<0.01
d 16–30				
ADFI (g/d)	62.04	66.67	1.78	0.06
ADG (g/d)	17.07^a^	13.55^b^	0.61	0.04
F/G	3.64^a^	4.92^b^	0.18	0.02
d 31–45				
ADFI (g/d)	60.05^a^	66.55^b^	0.70	<0.01
ADG (g/d)	22.44^a^	17.22^b^	0.62	0.01
F/G	2.68^a^	3.86^b^	071	<0.01

BW, body weight; ADG, average daily gain; ADFI, average daily feed intake; F/G, ADFI/ADG; SEM, standard error of means; d, day. ^1^IOFP, basal diet supplemented with IOFP 0.8% (w/w); Qinhuangdao Gaotong Biotech Co., Ltd., Changli, China. ^2^Control, basal diet. Different lowercase superscript letters indicate significant differences (*P* < 0.05). Mean values were calculated based on two cats per replicate and five replicates per group.

### 3.2 Effects of IOFP on the blood glucose level of cats

**Figure 1:**
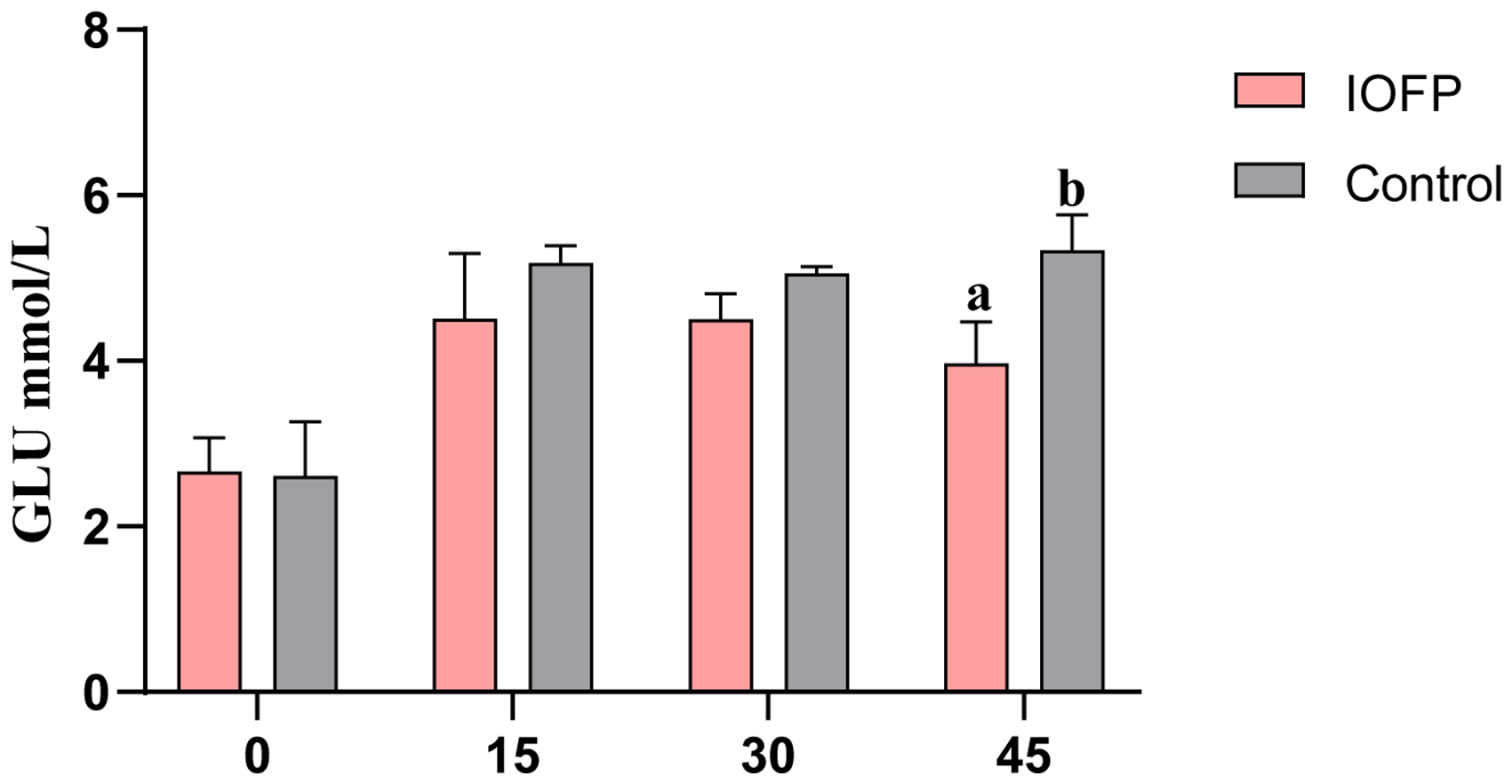
Effect of *Inonotus obliquus* fermentation product (IOFP) on the blood glucose level of the cats. Different lowercase letters indicate a significant difference (*p* < 0.05) between the IOFP and control groups. The error bars are based on the standard error of means.

Blood glucose levels of cats in both groups were measured every 15 days after 12 h of fasting. As depicted in Figure 1, at each time point, blood glucose levels in the IOFP group were lower than those in the control group, although the difference was not significant during the first 30 days (*p* > 0.05). These results indicate that IOFP can effectively reduce blood glucose levels in cats. Furthermore, cats in the IOFP group showed no un comfortable clinical signs, suggesting its relatively moderate hypoglycemic effect.

### 3.3 Effects of IOFP on the serum biochemical parameters of cats

**Figure 2:**
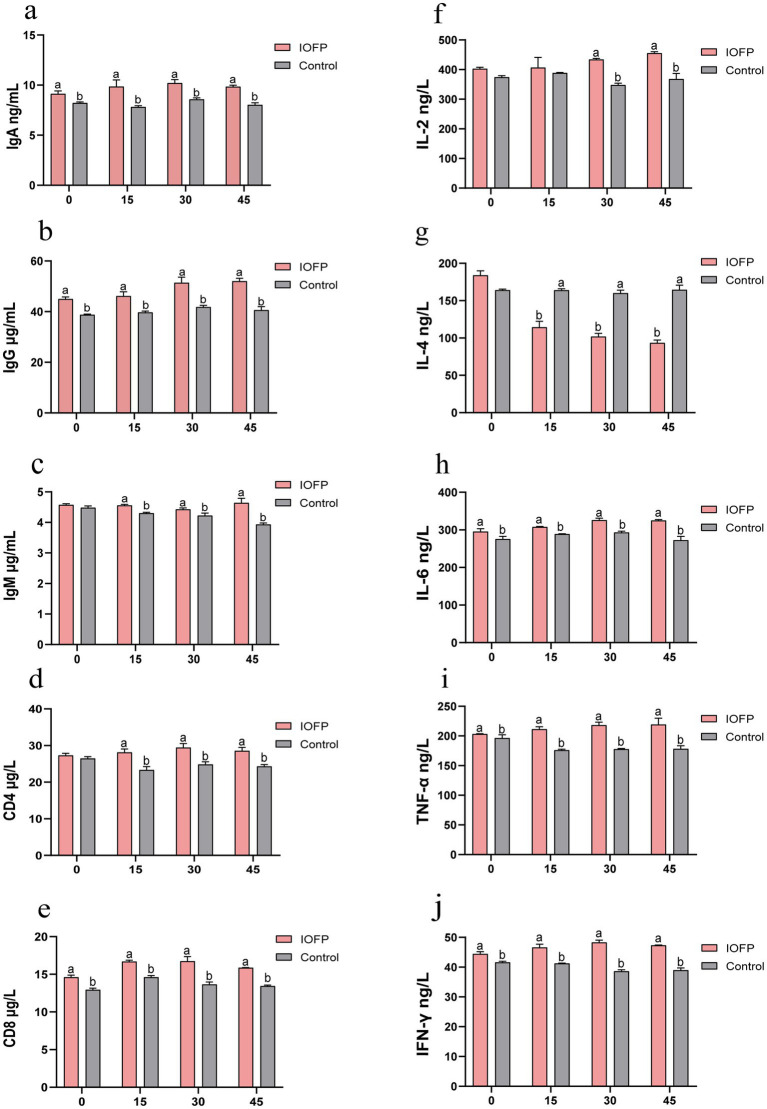
Effect of *Inonotus obliquus* fermentation product (IOFP) on serum biochemical indicators of cats. **(a)** Concentrations of IgA in the IOFP and control groups. **(b)** Concentrations of IgG in the IOFP and control groups. **(c)** Concentrations of IgM in the IOFP and control groups. **(d)** Concentrations of CD4 in the IOFP and control groups. **(e)** Concentrations of CD8 in the IOFP and control group. **(f)** Concentrations of IL-2 in the IOFP and control group. **(g)** Concentrations of IL-4 in the IOFP and control group. **(h)** Concentrations of IL-6 in the IOFP and control groups. **(i)** Concentrations of TNF-*α* in the IOFP and control groups. **(j)** Concentrations of IFN-*γ* in the IOFP and control groups. Different lowercase letters indicate a significant difference (*p* < 0.05) between the IOFP and control groups. The error bars are based on the standard error of means.

To determine the effects of IOFP on immune function in cats, relevant indicators were measured. Results showed that dietary supplementation with IOFP significantly increased the levels of IgA, IgM, and IgG (*p* < 0.05), suggesting that it induced a strong humoral immune response. Moreover, the concentrations of CD4^+^ and CD8^+^ T-cell subsets were significantly elevated, indicating a marked enhancement in cellular immunity. Furthermore, cytokine expression analysis revealed that the levels of IL-2, IL-4, and IFN-*γ* increased, while the concentration of TNF-*α* decreased (Figure 2). These findings revealed that dietary IOFP supplementation could enhance immune function and alleviate systemic inflammation in cats significantly.

### 3.4 Effects of IOFP on the intestinal morphology of cats

Morphology analysis of the small intestine revealed that IOFP supplementation significantly increased villus height and crypt depth across all three segments (Table 3), and subsequent V/C ratio were enhanced. Therefore, IOFP improved the development and mucosal morphology of intestine.

**Table 3:** Effect of *Inonotus obliquus* fermentation product (IOFP) on intestinal tissue morphology.

Intestinal segments	Item	IOFP^1^	Control^2^	SEM	*P-*value
Duodenum	Villi height, μm	698.54^a^	532.07^b^	1.73	<0.01
	Crypt depth, μm	409.53^a^	467.29^b^	2.44	0.01
	V/C	1.57^a^	1.28^b^	0.22	<0.01
Jejunum	Villi height, μm	758.87^a^	597.94^b^	0.50	<0.01
	Crypt depth, μm	391.30^a^	498.48^b^	0.99	<0.01
	V/C	1.92^a^	1.20^b^	0.10	<0.01
Ileum	Villi height, μm	294.17^a^	392.47^b^	3.24	<0.01
	Crypt depth, μm	277.57^a^	334.22^b^	1.44	0.03
	V/C	1.06^a^	1.19^b^	0.15	0.14

V/C, villi height to crypt depth; SEM, standard error of means. ^1^IOFP, basal diet supplemented with IOFP 0.8% (w/w); Qinhuangdao Gaotong Biotech Co., Ltd., Changli, China. ^2^Control, basal diet. Different lowercase superscript letters indicate significant differences (*P* < 0.05). The mean values were calculated based on two cats per replicate and five replicates per group.

### 3.5 Effects of IOFP on the gut microbiota of cats

**Figure 3:**
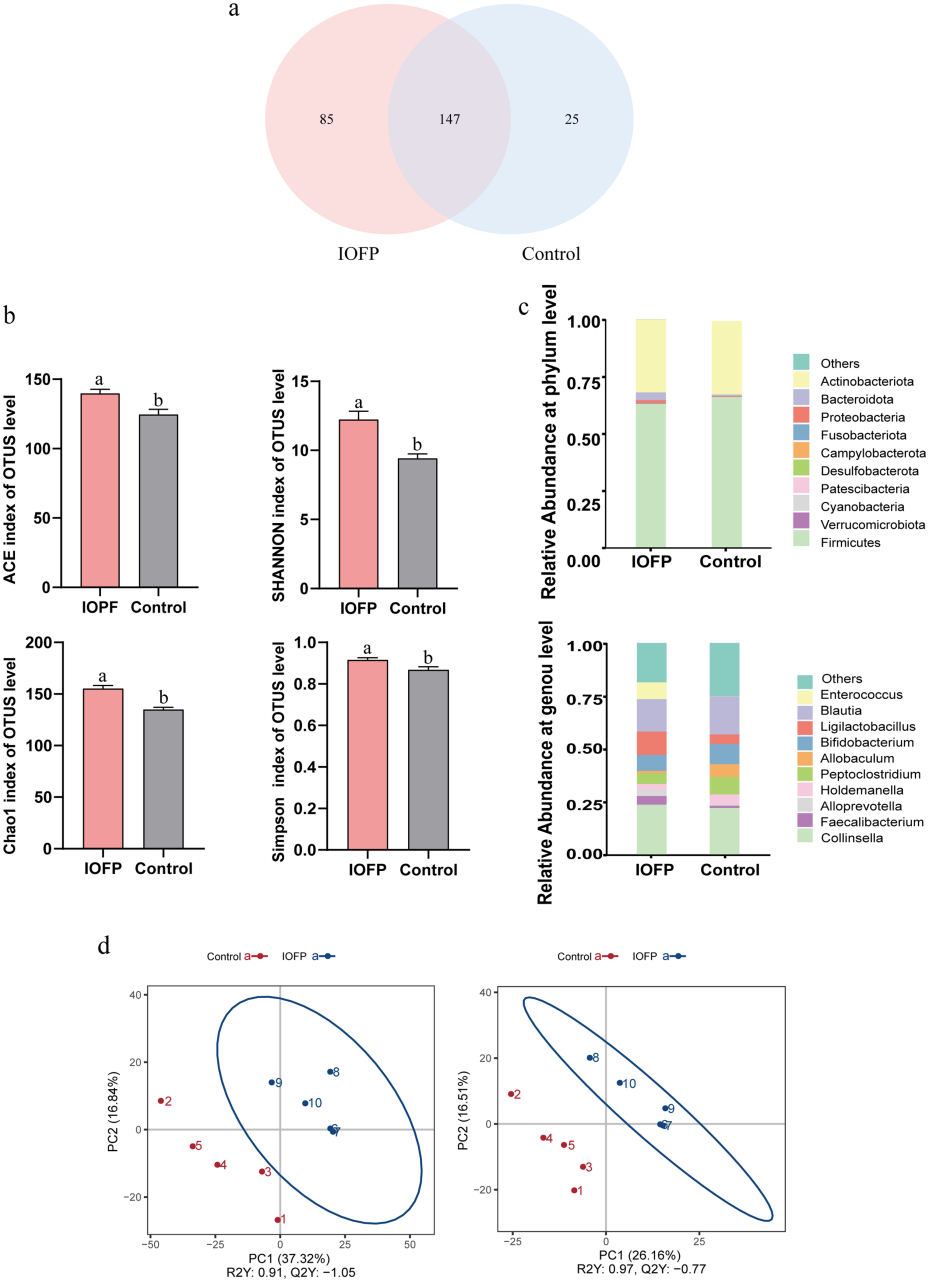
Effect of *Inonotus obliquus* fermentation product (IOFP) on the cecal microbiota of cats. **(a)** Venn diagram of OTU between the IOFP and control groups. **(b)** Analysis of alpha diversity between the IOFP and control groups. **(c)** Gut microbiota composition analysis at the phylum and genus level between the IOFP and control groups. **(d)** Composition of cecal metabolites under positive (left panel) and negative (right panel) ions of liquid chromatography–tandem mass spectrometry (LC–MS/MS) in the IOFP and control groups, as analyzed by partial least squares-discriminant analysis (PLS-DA). Different lowercase superscript letters indicate significant differences (*p* < 0.05). The error bars are based on the standard error of means.

As illustrated in Figures 3A, 232 and 172 OTUs were identified in the guts of the IOFP and control groups, respectively, of which 147 were shared. Subsequently, the *α*-diversity of the microbiota was evaluated. The results indicated that the Chao1, ACE, Shannon, and Simpson indices were significantly higher in the IOFP group than those in the control group (Figure 3B).

Furthermore, the composition of the intestinal microbiota was analyzed. Under normal conditions, Actinobacteria and Bacteroidota were predominant in the cats at the phylum level. As IOFP feeding for 45 days, the relative abundances of the two phyla increased significantly (*p* < 0.05), whereas that of Firmicutes decreased (*p* < 0.05). Therefore, the Firmicutes-to-Bacteroidota ratio was significantly lower in the IOFP group, which suggesting a potentially beneficial effect on weight management in cats. At the genus level, IOFP significantly increased the relative abundances of *Collinsella*, *Enterococcus*, *Alloprevotella*, and *Ligilactobacillus*, while decreased those of *Blautia*, *Peptoclostridium*, *Holdemanella*, and *Allobaculum* (Figure 3c).

### 3.6 Effects of IOFP on the intestinal metabolites of cats

Further studies were performed to determine the changes in intestinal metabolites. Partial least squares discriminant analysis (PLS-DA) showed that the R^2^Y values for the positive and negative ion modes were 0.91 and 0.97, respectively (Figure 3d). The two-dimensional score plot showed a clear separation of samples from the IOFP and control groups, indicating a significant alteration in metabolite composition. Of the top 10 significantly different metabolites between the two groups, four (goniothalamusin, 11Z,14Z,17Z,20Z,23Z-hexacosapentaenoic acid, phaffiaol, and 1-Thiocyanato-4-(methylthio) butane) increased and six (purine, oleandrin, decanoic acid, spermic acid 2, sedumoside A2, and linalyl acetate) decreased, respectively (Table 4).

**Table 4:** Differential metabolites in partial least squares-discriminant analysis (PLS-DA).

NO	Metabolite^1^	Mode^2^	FC	*P*-value
1	Goniothalamusin	+	23.62	0.027
2	Purine	+	0.08	0.002
3	Decanoic acid	−	0.15	0.010
4	Spermic acid 2	+	0.18	0.045
5	11Z,14Z,17Z,20Z,23Z-hexacosapentaenoic acid	+	5.54	0.031
6	Oleandolide	+	0.19	0.038
7	Phaffiaol	−	4.87	0.025
8	Sedumoside A2	−	0.23	0.018
9	Linalyl acetate	+	0.24	0.013
10	1-Thiocyanato-4-(methylthio)butane	−	4.08	0.050

NO, number; FC, fold change. ^1^Metabolite, top 10 metabolites with the highest fold change between treated and untreated groups. ^2^Mode, positive (+) and negative (−) ions of LC–MS/MS in PLS-DA. Mean values are based on one cat per replicate and five replicates per group.

### 3.7 Correlation analysis between gut microbiota and metabolites

**Figure 4:**
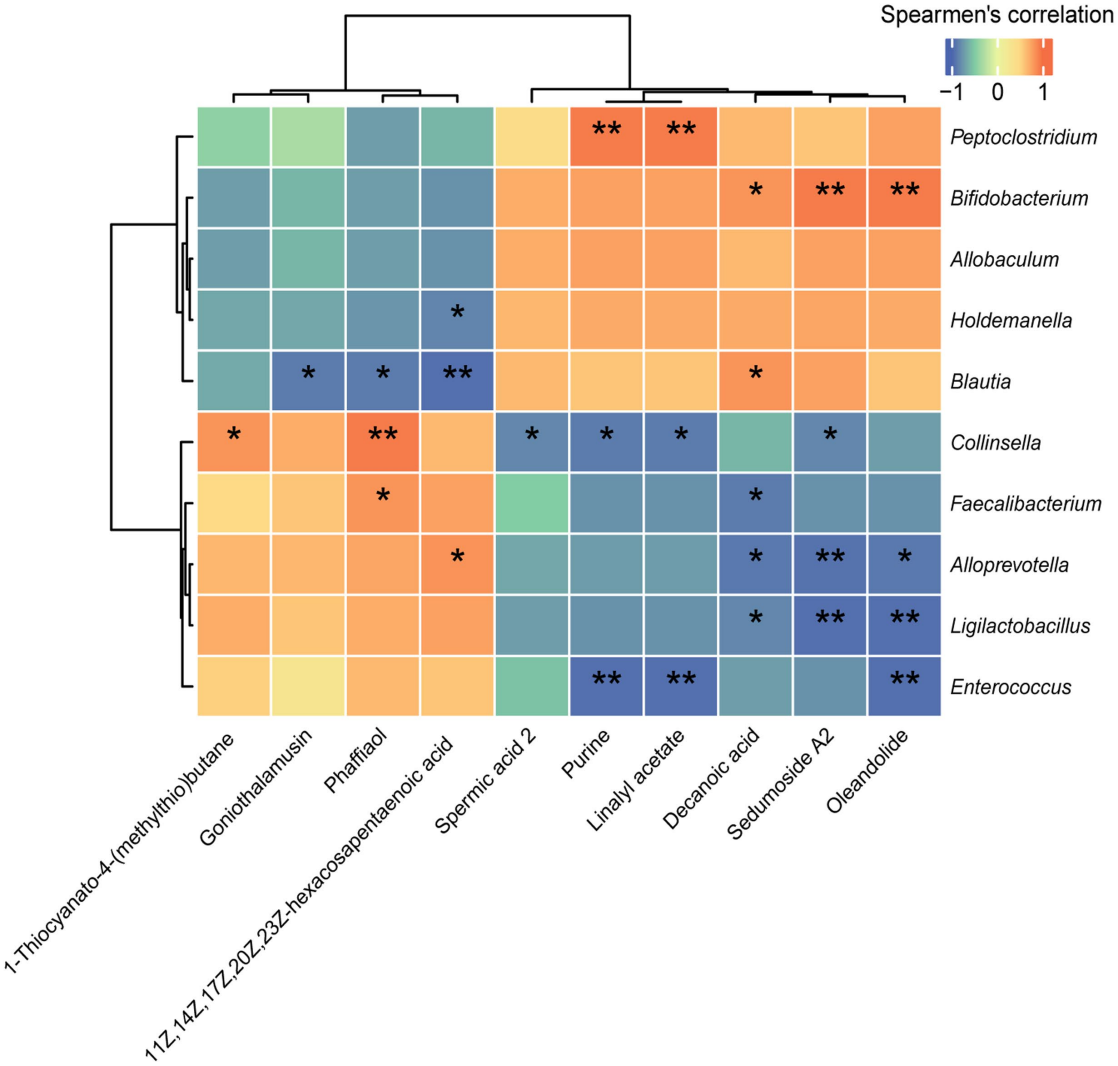
Correlational analysis of differential metabolites and microbes. The top 10 differential metabolites regarded as fold change (FC) and microbes regarded as *p*-value at the genus level were selected to conduct correlational analysis using Pearson’s method. Red and blue ellipses mean positive and negative correlation, respectively. *means significant correlations (*p* < 0.05) between the metabolite and the microbe. **means significant correlations (*p* < 0.01) between the metabolite and the microbe.

Pearson correlation analysis was performed between the top 10 differential microbiota (genus) and metabolites. Results demonstrated that the upregulated metabolites in the IOFP group were positively correlated with *Collinsella*, *Ligilactobacillus*, *Alloprevotella*, *Faecalibacterium*, and *Enterococcus* and negatively correlated with *Bifidobacterium*, *Blautia*, *Peptoclostridium*, *Holdemanella*, and *Allobaculum*. By contrast, the downregulated metabolites were positively correlated with *Bifidobacterium, Blautia*, *Peptoclostridium*, *Holdemanella,* and *Allobaculum* and negatively correlated with the other five genera (Figure 4).

### 3.8 Effects of IOFP on the intestinal SCFAs of cats

**Figure 5:**
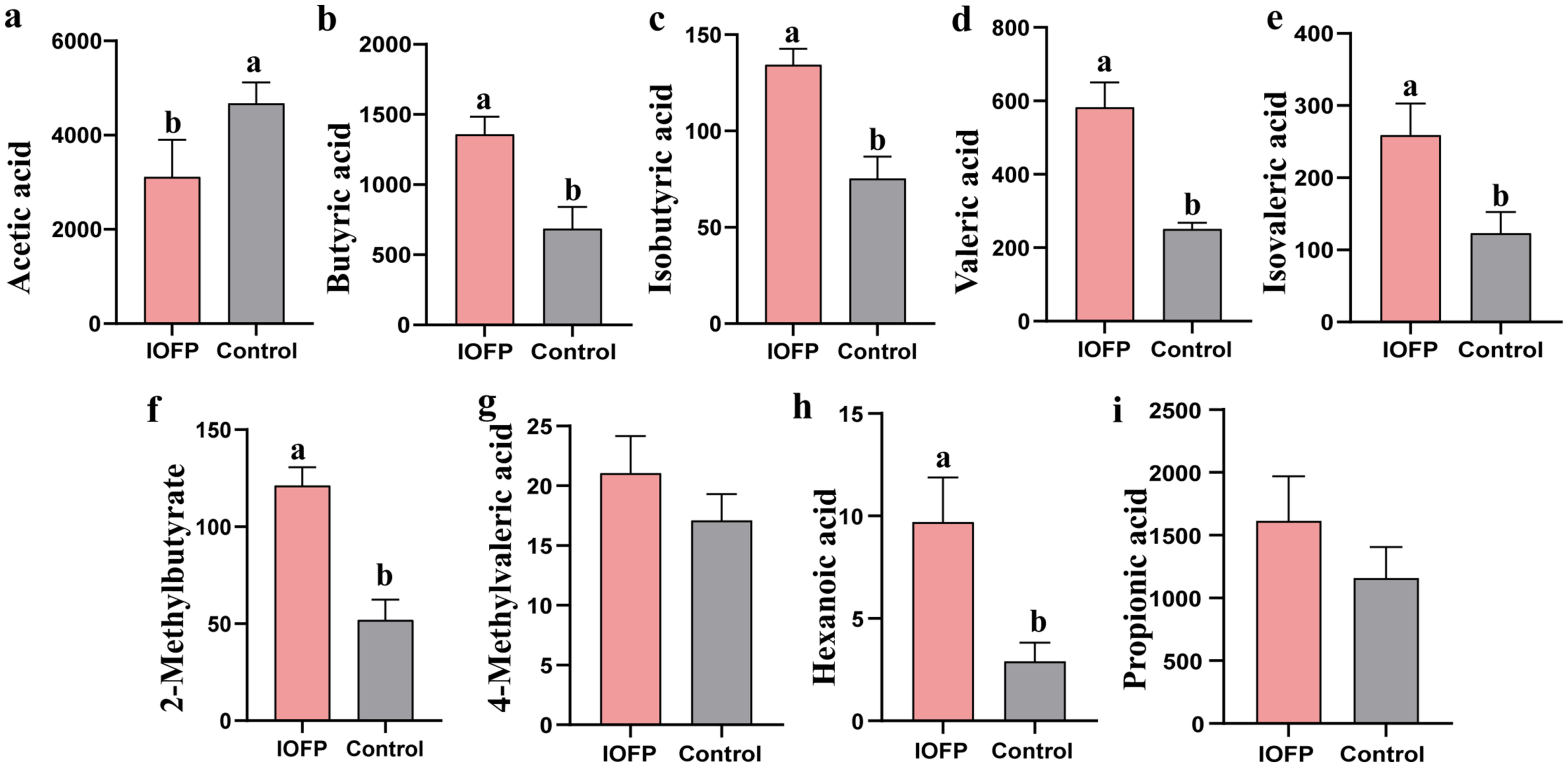
Effect of *Inonotus obliquus* fermentation product (IOFP) on intestinal short-chain fatty acids (SCFAs) of cats. **(a)** Contents of acetic acid in the IOFP and control groups. **(b)** Contents of butyric acid in the IOFP and control groups. **(c)** Contents of isobutyric acid in the IOFP and control groups. **(d)** Contents of valeric acid in the IOFP and control groups. **(e)** Contents of isovaleric acid in the IOFP and control groups. **(f)** Contents of 2-methylbutyrate in the IOFP and control groups. **(g)** Contents of 4-methylvaleric acid in the IOFP and control groups. **(h)** Contents of hexanoic acid in the IOFP and control groups. **(i)** Contents of propionic acid in the IOFP and control groups. Different lowercase letters indicate a significant difference (*p* < 0.05) between the IOFP and control groups. The error bars are based on the standard error of means.

Finally, the concentrations of SCFAs in the cecal contents were estimated. As depicted in Figure 5, the concentrations of all detected SCFAs (except acetate) were higher in the IOFP group than those in the control group. Specifically, the levels of butyrate, isobutyrate, valerate, isovalerate, 2-methylbutyrate, and caproate were significantly increased (*p* < 0.05), whereas that of propionate was not significant.

## 4 Discussion

With the continuous upgrading of attention to pet health, wishes of people to control and treat disease using natural or green biological agents are becoming stronger. However, related antibiotic alternatives are scare. In recent years, several plant ingredients demonstrated their physiological functions on laboratory level in cats or dogs, such as fucoidan, polyphenol and flavone, which could improved growth performance, immune function and intestine health (20, 21). *I. obliquus* is a medicinal and edible fungus rich in several bioactive compounds, such as polysaccharides, polyphenols, inositol, inotodiol, sterols, and triterpenoids. These ingredients confer it diverse physiological functions, including antitumor, antioxidant, hypoglycemic, immunomodulatory, antibacterial, and antiviral effects (9, 22, 23). Compared with plant ingredients mentioned above, IOFP had been supplemented in diets of several animals (chicken, duck, rabbit and pig), and results had shown beneficial effects on growth performance and immune function, suggesting that it was not only an effective feed additive and immunomodulator but also a potential alternative to antibiotics (12–14, 24). However, whether similar benefits could be achieved by adding IOFP to the diet of domestic cats remains unclear. In this study, supplementing the diet of weaned kittens with 0.8% (w/w) IOFP significantly increased ADG and feed conversion efficiency throughout the trial, which was similar with results obtained in meat rabbits and dogs (14, 25). A healthy intestine is the foundation for growth improvement. Dietary 0.2% methylsulfonylmethane supplementation has been reported to improve growth performance by shifting the physiological state (26). Similarly, ferulic acid added to the diet has been found to significantly enhance intestinal function and growth performance by increasing gut microbiota diversity and improving intestinal health in broilers (27). This study evaluated intestinal morphology and the gut microbiota in both groups of cats. Following IOFP supplementation, villus height, crypt depth, and the V/C ratio improved across different sections of the small intestine, indicating enhanced intestinal digestive and absorptive functions. These findings also partly explained the rapid weight gain and improved feed conversion observed in this research. Subsequent analysis of the gut microbiota revealed that IOFP supplementation modulated microbial diversity and composition. Notably, the relative abundance of Bacteroidota increased significantly. This bacterial group has a strong ability to degrade polysaccharides, allowing more nutrients to be absorbed and promoting organism development (28). Furthermore, the abundance of many beneficial bacteria at the genus level increased, such as *Lactobacillus* and *Enterococcus*. They could secrete substantial digestive enzymes to assist in nutrient absorption (29, 30). In addition, altered metabolites derived from changes in microbial composition exerted significant effects on improving growth performance. For example, SCFAs contributed to nutrient digestion and absorption by lowering gastrointestinal pH, enhancing pancreatic function, and promoting intestinal mucosal morphology (31). In summary, the improved intestinal morphology, microbiota, and metabolites benefited the growth performance of cats, which increased considerably.

High blood glucose level is one of the most common metabolic diseases in older cats (32). However, safe and effective medications are not enough. Previous studies have shown that *I. obliquus* possess significant hypoglycemic activity (33). Therefore, IOFP was continuously supplemented in the diet of cats for 45 days, and result displayed the reduction of fasting blood glucose levels without adverse hypoglycemic reactions. In recent years, the role of the “gut microbiota–metabolite–host” axis in the regulation of glucose metabolism has attracted immense attention (34, 35). Several probiotics could improve glucose metabolism via various pathways. *Lactobacillus plantarum* alleviates insulin resistance by increasing the production of SCFAs (36), whereas *Lactobacillus rhamnosus* decreases fasting blood glucose levels by reducing the release of endotoxins into the bloodstream (37). In this study, abundance of beneficial bacteria and SCFAs increased, many of which had the potential to stimulate intestinal L cells to secrete glucagon-like peptide-1, which improving insulin secretion and sensitivity (38). Therefore, gut microbiota and beneficial metabolites optimization may be a key mechanism by which IOFP regulates blood glucose levels. However, because obtaining sufficient numbers of elderly cats for blood glucose monitoring or experimental research at the same time is difficult, only a preliminary study of the hypoglycemic effect of IOFP in kittens was conducted. Research on elderly cats will be conducted in the future.

Previous studies have shown that the oral administration of IOFP can significantly improve the development of immune organs, immunoglobulin levels (such as IgG and IgA), cytokine levels (IL-2, IFN-*γ*, etc.), and overall immune status in mammals (13, 14). Similar results were observed when IOFP was fed to the cats, with notable enhancement of humoral immunity, cellular immunity, and cytokine expression. The intestine is the largest immune organ in the body, and the gut microbiota and its metabolites are the critical link between nutritional interventions and host immune responses (39). In this study, composition of bacteria and metabolites were changed significantly, as IOFP supplemented in the diet. Relative abundance of several pathogenic bacteria associated with mucosal damage and intestinal inflammation decreased, while bacteria with immunomodulatory functions enriched, such as *Lactobacillus*, *Collinsella*, *Prevotella*, and *Faecalibacterium* (40–42). Moreover, microbiota-derived SCFAs, goniothalamusin and sedumoside A2, which could enhance immune response and maintain immune homeostasis, were significantly increased (43–45). Therefore, the enhancement of immune function in cats can be attributed to the optimization of beneficial microbiota and corresponding metabolites by IOFP.

Pearson correlation analysis revealed significant associations between differentially expressed metabolites and specific bacteria, and most metabolites were coregulated by multiple bacteria. Therefore, IOFP likely improved the physiological function of cats by regulating the composition and abundance of the gut microbiota. This mechanism of action may be derived from changes in metabolite utilization patterns caused by alterations in the gut microbiota, and this relationship will be determined in our further studies.

## 5 Conclusion

In summary, dietary supplementation with 0.8% (w/w) IOFP improved the growth performance, gut morphology, immune function and blood glucose level. Moreover, compositions of gut microbiota were also optimized and derived metabolites and SCFAs were regulated by various bacteria. Thus, IOFP could serve as a safe and effective functional agent and contribute to disease prevention in cats.

## Data Availability

The datasets presented in this study can be found in online reposi-tories. The 16S rRNA gene sequencing and metabolites data are available from NCBI (PRJNA1428769 (https://dataview.ncbi.nlm.nih.gov/object/PRJNA1428769?reviewer=rs09fb50l1cq7onkn5hnek044q)) and MetaboLights (MTBLS13935 (https://www.ebi.ac.uk/metabolights/MTBLS13935)).
